# EEG Feature Selection via Stacked Deep Embedded Regression With Joint Sparsity

**DOI:** 10.3389/fnins.2020.00829

**Published:** 2020-08-06

**Authors:** Kui Jiang, Jiaxi Tang, Yulong Wang, Chengyu Qiu, Yuanpeng Zhang, Chuang Lin

**Affiliations:** ^1^Department of Medical Informatics of Medical (Nursing) School, Nantong University, Nantong, China; ^2^Shenzhen Institutes of Advanced Technology, Chinese Academy of Sciences, Shenzhen, China

**Keywords:** brain-computer interface, feature selection, stacked deep structure, stacked generalized principle, EEG

## Abstract

In the field of brain-computer interface (BCI), selecting efficient and robust features is very seductive for artificial intelligence (AI)-assisted clinical diagnosis. In this study, based on an embedded feature selection model, we construct a stacked deep structure for feature selection in a layer-by-layer manner. Its promising performance is guaranteed by the stacked generalized principle that random projections added into the original features can help us to continuously open the manifold structure existing in the original feature space in a stacked way. With such benefits, the original input feature space becomes more linearly separable. We use the epilepsy EEG data provided by the University of Bonn to evaluate our model. Based on the EEG data, we construct three classification tasks. On each task, we use different feature selection models to select features and then use two classifiers to perform classification based on the selected features. Our experimental results show that features selected by our new structure are more meaningful and helpful to the classifier hence generates better performance than benchmarking models.

## Introduction

**FIGURE 1:**
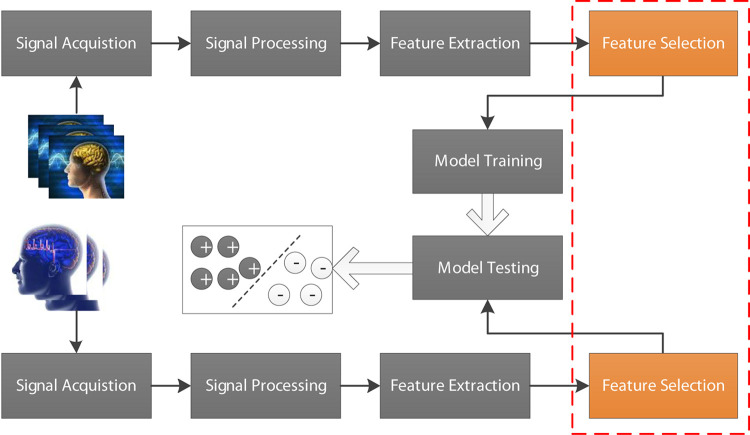
EEG-based AI-assisted diagnosis flowchart containing signal acquisition, signal processing, feature extraction, feature selection, and model training and testing.

Electroencephalogram (EEG) as a biomarker plays an important role in the brain-computer interface (BCI) (Wang et al., 2013; Zheng, 2017; Mammone et al., 2019; Nakamura et al., 2020). For example, EEG signals are often used to determine the presence and type of epilepsy in clinical diagnosis (Rieke et al., 2003; Yetik et al., 2005; Adeli et al., 2007; Lopes da Silva, 2008; Coito et al., 2016; Parvez and Paul, 2016; Peker et al., 2016; Panwar et al., 2019). In recent years, with the rapid development of artificial intelligence technology, AI-assisted diagnosis has attracted more and more attention and achieved unprecedented success in many scenarios including BCI (Agarwal et al., 2018; Wu et al., 2018). In general, a standard EEG-based AI-assisted diagnosis flowchart is illustrated in Figure 1, which contains signal acquisition, signal processing, feature extraction, feature selection and model training and testing. As we know that original features extracted from EEG signals cannot be directly used for model training because they are often represented in very high-dimensional feature space. Therefore, feature selection is usually performed before model training. In this study, we focus on how to selection effective features to guarantee high-efficiency AI-assisted clinical diagnosis.

To the best of our knowledge, most of the existing feature selection models belong to one of three main catalog, i.e., filter, embedded, and wrapper (Visalakshi and Radha, 2014; Ang et al., 2016; Shah and Patel, 2016; Saputra Rangkuti et al., 2018). In filter models, feature selection depends on the intrinsic properties and the relevancies existing among features. That is to say, filter models are independent of classifiers. Some of the most commonly-used filter models include mRMR (Peng et al., 2005), F-statistic (Habbema and Hermans, 1977), Chi-square and information gain (Raileanu and Stoffel, 2004), *t*-test (Raileanu and Stoffel, 2004) and Relief (Kira and Rendell, 1992), etc. All of them perform feature selection by making use of global statistical information such as the relevance/sensitivity/correlation of a feature w.r.t the class label distribution of the data. In wrapper models, feature selection is around classifiers providing them subsets of features and receiving their feedback. Different from filter models, wrapper models are tightly coupled with a specific classifier. Some representative models include CFS (Hall and Smith, 1999) and RFE-SVM (Guyon et al., 2002), etc. In embedded models, feature selection is considered as an optimization problem and integrating into a specific classifier so that the selected features have a seductive effect on the corresponding classification task. For example, Nie et al. (2010) integrated *l*_2_, _1_-norm into a robust loss function and proposed an efficient and robust model (renamed as E-JS-Regression) to perform feature selection. Their experimental results on several biomedical data indicated that E-JS-Regression won better performance than both filter models and wrapper models.

In ensemble learning (Webb and Zheng, 2004; Minku et al., 2010; Chen et al., 2017; Liu et al., 2019; Zhu et al., 2020), stacking is a popular classifier combination strategy which takes the outputs of other classifier as input to train a generalizer. In Wolpert (1992) proposed the stacked generalization principle which indicated that the outputs can help to open the manifold of data distribution. In our previous work (Zhang et al., 2018), we made use of this principle and proposed a deep TSK fuzzy system. Therefore, in this study, based on this principle and by taking E-JS-Regression as the basic component, we will construct a layer-by-layer stacked deep structure for feature extraction. The new model is termed as SDE-JS-Regression. In SDE-JS-Regression, each component is connected in a layer-by-layer manner, the output of the previous layer is transformed by random projection as a random shift and then added into the input space. The new input space is considered as the input to the next component. In such a way, the manifold in the training space is continuously opened. The contribution of this study is summarized as follows:

(i) Based on E-JS-Regression proposed by Nie et al. we construct a stacked deep structure for feature selection in a layer-by-layer manner so as to add random projections into the original features so that the manifold structure existing in the original feature space is continuously opened in a stacked way. Therefore, according to the stacked generalized principle, the original input feature space becomes more linearly separable.
(ii) We build three classification tasks from epilepsy EEG data provided by the University of Bonn and introduce different kinds of feature selection methods to demonstrate the promising performance of our proposed method.

## Data and Methods

### Data

**FIGURE 2:**
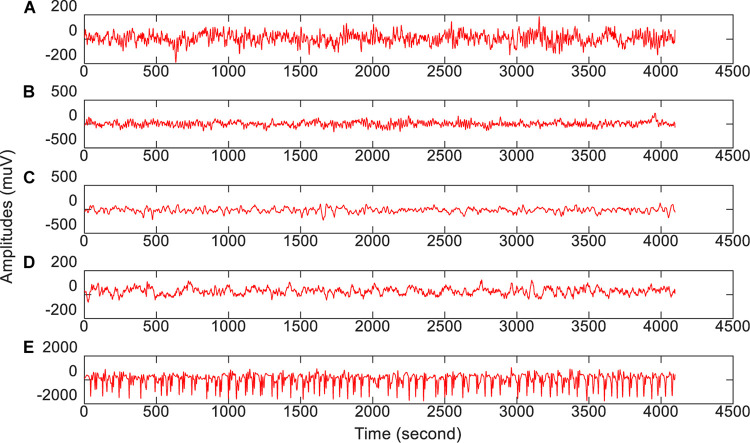
The amplitude of one subject in each group during the collection procedure. From top to bottom corresponds to **(A–E)**, respectively.

The epilepsy EEG data downloaded from the University of Bonn will be used to evaluate our proposed feature selection model. This dataset consists of 5 groups of subsets (from group A to group E), where each group is composed of 100 single channel EEG segments during 23.6 s duration. Segments in group A and group B are collected from 5 healthy subjects, while segments in the rest groups are collected from epileptics. Table 1 lists the data structure and collection conditions. Additionally, Figure 2 (Zhang et al., 2020) illustrates the amplitudes during the collection procedure of one subject in each group.

**TABLE 1:** Data structure and collection conditions of epilepsy EEG segments.

**Volunteers**	**Groups**	**#Channels**	**#Features**	**Collection conditions**
Health	A	100	4097	Signals were recorded when volunteer subjects were relaxed in awaken state with eyes open.
	B	100	4097	Signals were recorded when volunteer subjects were relaxed in awaken state with eyes closed.
Epileptic	C	100	4097	Signals were recorded from the hippocampal formation of the opposite hemisphere of brain.
	D	100	4097	Signals were recorded within epileptogenic zone during seizure free intervals.
	E	100	4097	Signals were recorded during seizure activity.

### Methods

In this section, we will give technical details of our proposed method including its framework, optimization, and algorithm steps. Before we do that, we first summarize the following used notations and some commonly-used definitions.

#### Notations and Definitions

We use **X** = [**x**_1_,**x**_2_,…,**x**_*n*_] ∈ *R*^*d*×*n*^ and **Y** = ^[**y**_1_,**y**_2_,…,**y**_*n*_]*T*^ ∈ *R*^*n*×*c*^ to represent a training set, where **x**_*i*_ = ^[*x*_1_,*x*_2_,…,*x*_*d*_]*T*^ ∈ ^*Rd*^ represents a training sample and *y*_*i*_ ∈ ^*Rc*^ is the corresponding label vector of **x**_*i*_, 1≤*i*≤*n*. For matrix **B**, we use *b*_*ij*_ to represent its element in the *i*-th row and *j*-th column, ^*bi*^ and *b*_*j*_ to represent its *i*-th row and *j*-th column, respectively. The *l*_2_,_1_-norm of matrix **B** is defined as:

(1) $$∥{\text{B}}_{2,1}∥=\sum_{i=1}^{n}{\left(\sum_{j=1}^{m}{\text{b}}_{i\,j}^{2}\right)}^{1/2}=\sum_{i=1}^{n}∥{\text{b}}_{2}^{i}∥$$

#### Structure of SDE-JS-Regression

**FIGURE 3:**
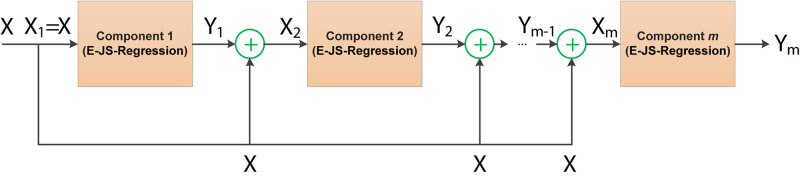
Stacked deep structure of SDE-JS-Regression.

In Nie et al. (2010) proposed an efficient and robust embedded regression model for feature selection via joint *l*_2_,_1_-norm sparsity (simplified as E-JS-Regression). Since *l*_2_-norm based loss function is sensitive to outlies, they used a *l*_2_,_1_-norm based loss function to remove outlies. Additionally, they also used a *l*_2_,_1_-norm to regularize the transformation matrix to select features with joint sparsity. That is to say, each feature either has small scores for all samples or has large scores for all samples. The objective function is defined as:

(2) $$\min_{W}J\,\left(W\right)={∥{\text{X}}^{T}\,\text{W}-\text{Y}∥}_{2,1}+\theta \,{∥W∥}_{2,1}$$

where θ is the regularized parameter, **W** ∈ *R*^*d*×*c*^. The stacked generalized principle as an ensemble learning strategy can provide an efficient way for model combination. Although the stacked generalized principle is not as widely used as boosting and bagging, its great innovation has been successful in many application scenarios. In this study, we take E-JS-Regression as a basic component to construct a stacked deep embedded regression model for EEG feature selection. Figure 3 illustrates the stacked deep structure of our proposed model.

The stacked deep structure is composed of *m* basic components linked in a layer-by-layer manner. To be specific, when the first component is fixed, the input to the subsequent components consists of two parts: the original input features and the output of the previous layer/component. How to fuse these two parts is very important in this study. Referring to the stacked generalized principle, we randomly generate a project of the output of the previous layer as a random shift and then integrate the random shift into the original input features. Therefore, the input of component *s* (1 < *s*≤*m*) **X**_*s*_ can be obtained by the following equation,

(3) $${\text{X}}_{s}^{T}={\text{X}}^{T}+\sigma \,{\text{Y}}_{s-1}\,\text{Z}$$

where **Z** ∈ *R*^*c*×*d*^ is a random projection matrix in which each element is in the range of [0, 1], σ is a positive regularized parameter. By virtue of this structure, all components (E-JS-Regression) are stacked and bridged by adding the original features to a continuous random shift to form the proposed feature selection model SDE-JS-Regression.

The benefits we inherit from the stacked deep structure lie in that the random projections added into the original features can help us to continuously open the manifold structure existing in the original feature space in a stacked way. With such benefits, the input feature space becomes more linearly separable.

#### Optimization of SDE-JS-Regression

By substituting (3) into (2), the optimization of SDE-JS-Regression can be considered as solving *m* subproblems. The *s*-th subproblem can be formulated as follows,

(4) $$\min_{W}J\,\left(W\right)=\frac{1}{\theta }\,{∥\left({\text{X}}^{T}+\sigma \,{\text{Y}}_{s-1}\,\text{Z}\right)\,\text{W}-\text{Y}∥}_{2,1}+{∥W∥}_{2,1}$$

which is equivalent to the following problem,

(5) $$\min_{W,Q}J\,\left(W\right)={∥Q∥}_{2,1}+{∥W∥}_{2,1}$$

(6) $$\text{s}.\text{t}.\left({\text{X}}^{T}+\sigma \,{\text{Y}}_{s-1}\,\text{Z}\right)\,\text{W}+\theta \,\text{Q}=\text{Y}$$

By equivalent transformation, we have:

(7) $$\min_{W,Q}J\,\left(W\right)={||\left[\begin{array}{c} \text{W}\\ \text{Q} \end{array}\right]||}_{2,1}$$

(8) $$\text{s}.\text{t}.\left[\begin{array}{cc} {\text{X}}^{T}+\sigma \,{\text{Y}}_{s-1}\,\text{Z}\,\theta \,\text{I} & \end{array}\right]\,\left[\begin{array}{c} \text{W}\\ \text{Q} \end{array}\right]=\text{Y}$$

where **I** ∈ *R*^*n*×*n*^ is a identity matrix. Let *h* = *n* + *d*, **K** = [**X**^*T*^ + σ**Y**_*s*−1_*Z*θ**I**] ∈ *R*^*n*×*h*^ and **V** = [**WQ**] ∈ *R*^*h*×*c*^, then the optimization problem in (7) can be updated as follows,

(9) $$\min_{V}J\,\left(V\right)={∥V∥}_{2,1}$$

(10) s.t.KV=Y

By introducing Lagrangian multiplies Δ, the corresponding Lagrangian function of (9) is formulated as follows,

(11) $$L\,\left(V\right)={∥V∥}_{2,1}-T\,r\,\left(\Delta^{T}\,\left(\text{KV}-\text{Y}\right)\right)$$

By setting the partial derivative of *L*(**V**) w.r.t **V** to 0, i.e.,

(12) $$\frac{\partial L\,\left(V\right)}{\partial \text{V}}=2\,\text{GV}-{\text{K}}^{T}\,\Delta =0$$

where **G** ∈ *R*^*h*×*h*^ is a diagonal matrix in which the *i*-th diagonal element is:

(13) $$g_{i\,i}=\frac{1}{2\,{∥v^{i}∥}_{2}}$$

Thus, by multiplying the two sides of (12) by **KG**^−1^, and making use of the constraint **KV** = **Y**, we have:

(14) $$\begin{array}{c} 2\,\text{KV}-{\text{KG}}^{-1}\,{\text{K}}^{T}\,\Delta =0\\ \Rightarrow 2\,\text{Y}-{\text{KG}}^{-1}\,{\text{K}}^{T}\,\Delta =0\\ \Rightarrow \Delta =2\,{\left({\text{KG}}^{-1}\,{\text{K}}^{T}\right)}^{-1}\,\text{Y} \end{array}$$

By substituting (14) into (12), we obtain **V** as:

(15) $$\text{V}={\text{G}}^{-1}\,{\text{K}}^{T}\,{\left({\text{KG}}^{-1}\,{\text{K}}^{T}\right)}^{-1}\,\text{Y}$$

#### Algorithm of SDE-JS-Regression

The detailed algorithm steps of SDE-JS-Regression are listed in Algorithm 1. When the transformation matrix **W** ∈ *R*^*d*×*c*^ is obtained by SDE-JS-Regression, we compute the sum of each column vector **w**_*j*_, then sort the elements in the final column vector from largest to smallest. In such a way, we obtain the feature ranking list, which can guide feature selection.

## Results

In this section, we will report our experimental settings and results.

### Setups

To fairly evaluate the feature selection performance of SDE-JS-Regression, we introduce serval types of feature selection models, i.e., E-JS-Regression (Nie et al., 2010), mRMR (Peng et al., 2005), RFE-SVM (Guyon et al., 2002), and Relief (Kira and Rendell, 1992) for benchmarking testing. A brief introduction of each benchmarking model is summarized as follows.

• E-JS-Regression: It is an embedded feature selection model and also the basic component of our proposed method. Its involved regularized parameter γ will be determined by 5-CV in our experiments.
• mRMR: It is a filtering feature selection model based on minimum redundancy and maximum relevancy. The redundancy is measured by mutual information.
• RFE-SVM: It is a wrapper feature selection model combining with the SVM classifier to achieve recursive feature elimination. Parameters in SVM are all determined by 5-CV.
• Relief: It is also a filtering feature selection model, which assigns a weight to each feature depending on the relevance between features and classes. The number of nearest neighbors is set to 10 in our experiments.

**Table:** 

**Algorithm 1:** SDE-JS-Regression
**Input**:
**X** = [**x**_1_,**x**_2_,…,**x**_*n*_] ∈ *R*^*d*×*n*^ and
**Y** = [**y**_1_,**y**_2_,…,**y**_*n*_]*T* ∈ *R*^*n*×*c*^
θ,σ*andm*
**Output**:
**W**
**Procedure:**
Set *t*←0
Initialize ^**G**(*t*)^ ∈ *R*^*h*×*h*^ as an identity matrix
Set *s*←1
Set **Y**_0_ = **0**

**Table:** 

Compute **K** = [**X**^*T*^ + σ**Y**_0_**Z**θ**I**] ∈ *R*^*n*×*h*^
**Repeat**

Compute **V**^(*t* + 1)^ = (**G**^(*t*)^)^−1^**K**^*T*^(**K**(**G**^(*t*)^)^−1^**K**^*T*^)^−1^**Y**
Compute **G**^(*t* + 1)^, where the *i*-th diagonal element is
$g_{i\,i}=\frac{1}{2\,{∥v^{i\,\left(t+1\right)}∥}_{2}}$
Set *t*←*t* + 1
**Until** |*J*^(*t* + 1)^(**V**)−*J*^(*t*)^(**V**)| <
Extract **W** from **V**
Compute **Y**_1_ = **X**^***T***^**W**
**For** *s* = 1*tom*

Set *t*←0
Initialize **G**^(*t*)^ ∈ *R*^*h*×*h*^ as an identity matrix
Randomly generate **Z** ∈ *R*^*c*×*d*^, where each element is in the
range of [0, 1]
Compute **K** = [**X**^*T*^ + σ**Y**_*s*_**Z**θ**I**] ∈ *R*^*n*×*h*^
**Repeat**

Compute **V**^(*t* + 1)^ = (**G**^(*t*)^)^−1^**K**^*T*^(**K**(**G**^(*t*)^)^−1^**K**^*T*^)^−1^**Y**
Compute **G**^(*t* + 1)^, where the *i*-th diagonal element
is $g_{i\,i}=\frac{1}{2\,v_{2}^{i\,\left(t+1\right)}}$
Set *t*←*t* + 1
**Until** |*J*^(*t* + 1)^(**V**)−*J*^(*t*)^(*V*)| < *ϵ*
Extract **W** from **V**
Compute **Y**_*s*_ = **X**^*T*^**W**
**End**

When the feature ranking list generated by each model is obtained, the Gaussian kernel based SVM (Chang and Lin, 2011) and Ridge regression (Ridge) (Yang and Wen, 2018) are employed to perform classification tasks. Based on the epilepsy EEG data shown in Table 1, we construct 3 classification tasks (see Table 2).

**TABLE 2:** Three classification tasks for selected features.

**No. of tasks**	**Volunteers**	**Groups**	**#Sizes**	**#Classes**	**Task description**
Task 1	Health	A, B	200	2	Classify healthy subjects to the eye-opening group and the eye-closing group.
Task 2	Epileptic	C, D and E	300	3	Classify epileptic subjects to non-seizure period, interseizure period and seizure period.
Task 3	Health/Epileptic	A, B, C, D and E	500	2	Classify all subjects into healthy group and epileptic group.

For each task, 75% samples are used for training and 25% samples are used for testing. Parameters (kernel width and slack variable) in the Gaussian kernel based SVM and the regularized parameter in Ridge are determined by 5-CV on the training set. Testing procedure is repeated 100 times and the average results in terms of *Accuracy* are recorded, where *Accuracy* is defined as the ratio of the number of correctly classified samples to the number of all samples.

### Experimental Results

**FIGURE 4:**
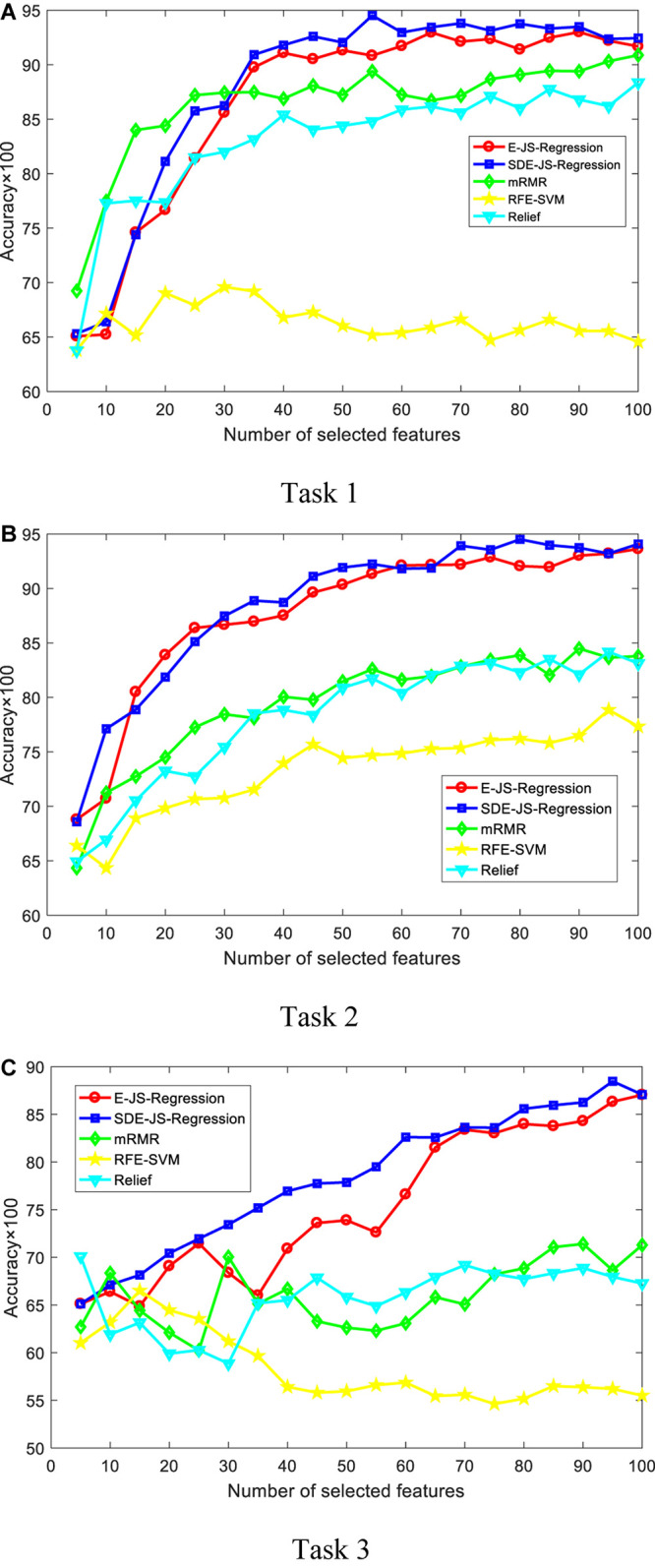
Classification performance by SVM. SDE-JS-Regression is our method. **(A)** Task 1. **(B)** Task 2. **(C)** Task 3.

**FIGURE 5:**
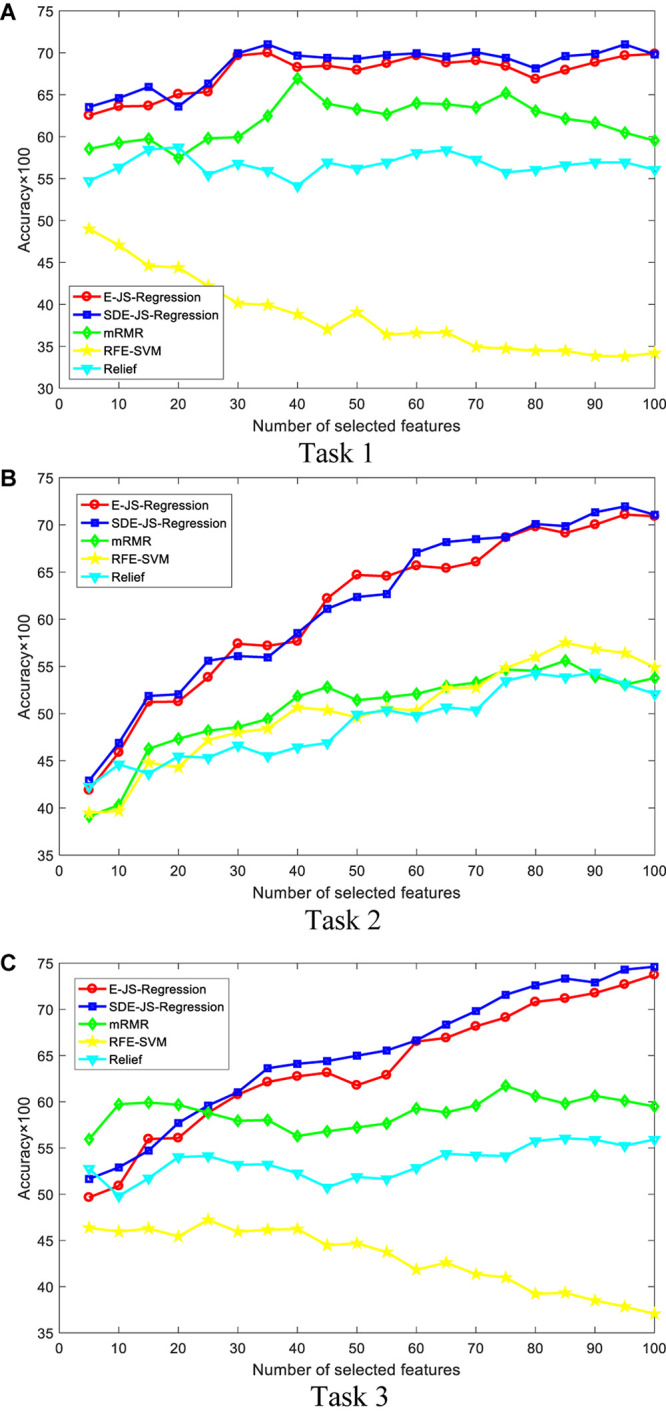
Classification performance by Ridge. SDE-JS-Regression is our method. **(A)** Task 1. **(B)** Task 2. **(C)** Task 3.

**FIGURE 6:**
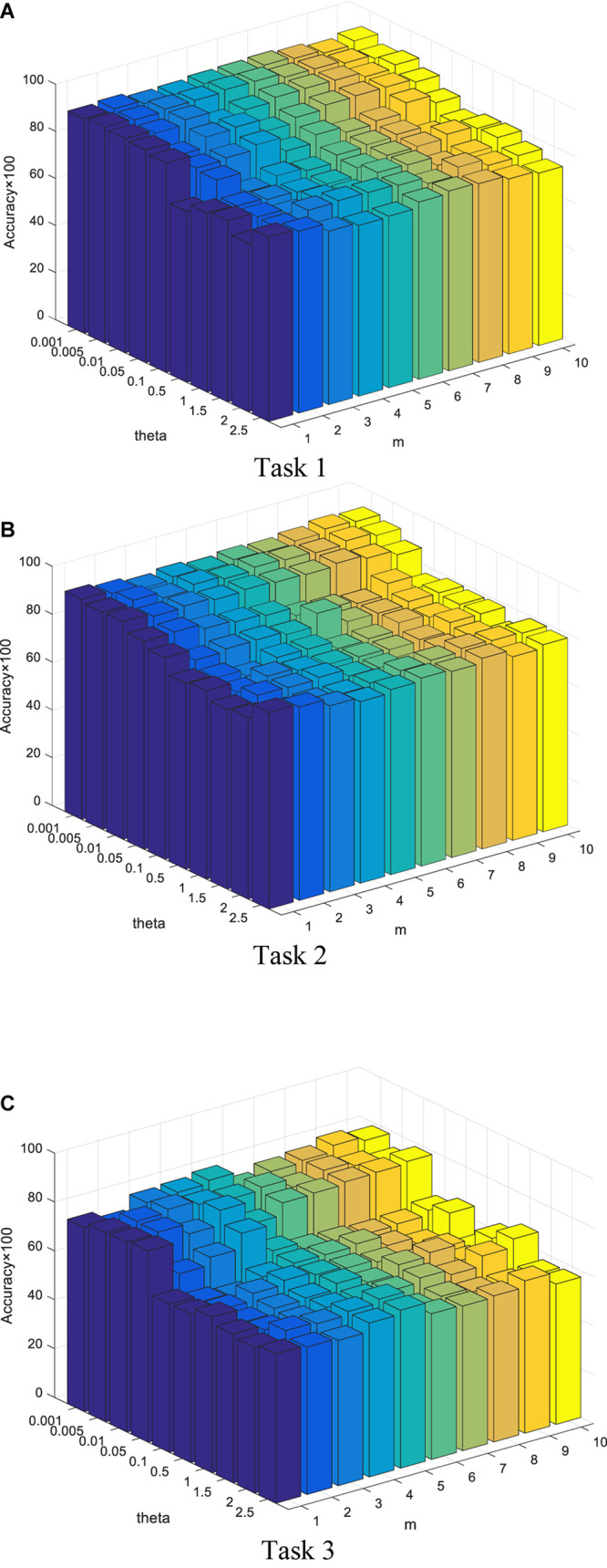
Parameter analysis w.r.t θ and *m*. Accuracy of each task is obtained on the top 55 features selected from the ranking feature list. **(A)** Task 1. **(B)** Task 2. **(C)** Task 3.

In this section, we report our experimental results from two main aspects, i.e., classification performance of selected features and the parameter analysis. Figures 4, 5 show the classification performance of five models with different numbers of features (from 5 to 100, step size is 5) selected from the corresponding ranking list. Figure 6 shows parameter analysis results w.r.t the regularized parameter θ and the number of components *m*, where θ is searched from the range [0.001, 0.005, 0.01, 0.05, 0.1, 0.5, 1, 1.5, 2, 2.5] and *m* is searched from the range [1–10]. Accuracy of each task is obtained on the top 55 features selected from the ranking feature list.

## Discussion

From the comparative results of three classification tasks shown in Figures 4, 5, we observe that SDE-JS-Regression performs better than the benchmarking models, especially mRMR, RFE-SVM, and Relief. On task 3, regardless of SVM or Ridge, SDE-JS-Regression always perform better than E-JS-Regression when the number of selected top features is bigger than 15. More characteristics are exhibited from the following aspects.

• From our experimental results, we find that features obtained from embedded feature selection models (SDE-JS-Regression and E-JS-Regression) are more inductive to the classifier than filter models (mRMR and Relief) and wrapper models (RFE-SVM). This is because embedded feature selection models minimize the classification training errors during the procedure of feature selection. Therefore, for our epilepsy classification tasks via EEG signals, embedded feature selection models are more suitable.
• On the three classification tasks, especially task 3, SDE-JS-Regression achieves better performance than E-JS-Regression, which indicates that our stacked deep structure can indeed help to select more classification addictive features and hence improve the classification performance. As we stated before, the benefits we inherit from the stacked deep structure lie in that the random projections added into the original features can help us to continuously open the manifold structure existing in the original feature space in a stacked way. With such benefits, the input feature space becomes more linearly separable.
• From Figure 5, with respect to θ, we observe that SDE-JS-Regression performs well in its range of [0.001, 0.05]. With the further increase of θ from 0.05 to 2.5, the classification performance begins to decrease. However, although the performance begins to decline when θ is in the range of [0.05, 2.5], the performance of SDE-JS-Regression does not show a significant change. Therefore, our proposed SDE-JS-Regression seems to be robust to θ. For our three EEG classification tasks, θ can be set from 0.001 to 0.05.
• The number of layers (components) in the structure of SDE-JS-Regression determines the number of random shifts added into the input feature space. As we can see from Figure 5 that “the more layers the better performance” is not holds. On the three tasks, 4–6 layers can guarantee a relatively good performance. Too many random shifts can lead to distribution distortion of the training set.

## Conclusion

In this study, we propose a feature selection model SDE-JS-Regression for AI-assisted clinical diagnosis through EEG signals. SDE-JS-Regression is quite different from the existing embedded models due to its stacked deep structure that is constructed in a layer-by-layer manner based on the stacked generalized principle. SDE-JS-Regression is derived from E-JS-Regression but performs better than E-JS-Regression since that random projections added into the original features can help us to continuously open the manifold structure existing in the original feature space in a stacked way so that the original input feature space becomes more linearly separable. We construct three classification tasks based on the selected features to evaluate the effectiveness of SDE-JS-Regression. Experimental results show that features selected by SDE-JS-Regression are more meaningful and helpful to the classifier hence generates better performance than benchmarking models. This study is not without limitations. For example, how to effectively determine the number of layers is very important. Therefore, in addition to CV, a new finding strategy will be desired in our coming studies.

## Data Availability Statement

Publicly available datasets were analyzed in this study. This data can be found here: http://www.meb.unibonn.de/epileptologie/science/physik/eegdata.html.

## Author Contributions

YZ, KJ, and CL designed the whole algorithm and experiments. JT, YW, and CQ contributed on code implementation. All authors contributed to the article and approved the submitted version.

## Conflict of Interest

The authors declare that the research was conducted in the absence of any commercial or financial relationships that could be construed as a potential conflict of interest.
